# Endomembrane-associated RSD-3 is important for RNAi induced by extracellular silencing RNA in both somatic and germ cells of *Caenorhabditis elegans*

**DOI:** 10.1038/srep28198

**Published:** 2016-06-16

**Authors:** Rieko Imae, Katsufumi Dejima, Eriko Kage-Nakadai, Hiroyuki Arai, Shohei Mitani

**Affiliations:** 1 Department of Physiology, Tokyo Women’s Medical University School of Medicine, Tokyo, Japan; 2 Graduate School of Pharmaceutical Science, University of Tokyo, Tokyo, Japan; 3 Tokyo Women’s Medical University Institute for Integrated Medical Sciences, Tokyo, Japan

## Abstract

RNA silencing signals in *C. elegans* spread among cells, leading to RNAi throughout the body. During systemic spread of RNAi, membrane trafficking is thought to play important roles. Here, we show that RNAi Spreading Defective-3 (*rsd-3*), which encodes a homolog of epsinR, a conserved ENTH (epsin N-terminal homology) domain protein, generally participates in cellular uptake of silencing RNA. RSD-3 is previously thought to be involved in systemic RNAi only in germ cells, but we isolated several deletion alleles of *rsd-3*, and found that these mutants are defective in the spread of silencing RNA not only into germ cells but also into somatic cells. RSD-3 is ubiquitously expressed, and intracellularly localized to the *trans*-Golgi network (TGN) and endosomes. Tissue-specific rescue experiments indicate that RSD-3 is required for importing silencing RNA into cells rather than exporting from cells. Structure/function analysis showed that the ENTH domain alone is sufficient, and membrane association of the ENTH domain is required, for RSD-3 function in systemic RNAi. Our results suggest that endomembrane trafficking through the TGN and endosomes generally plays an important role in cellular uptake of silencing RNA.

In multicellular organisms, cells communicate with each other through various signaling molecules, including proteins and lipids, to maintain tissue homeostasis and regulate growth and differentiation. Growing evidence indicates that RNA also function as an intercellular signaling molecule^1^,^2^. For example, extracellular RNA has been detected in various body fluids of mammals^3^, and some microRNAs are actually transported between cells and exert gene silencing^4^,^5^. Currently, the molecular mechanism of RNA transport between cells and the biological significance of extracellular RNA remain largely unknown.

In some animals, including *C. elegans*, introduction of double-stranded RNA (dsRNA) into cells induces RNA interference (RNAi) not only in the cells, but also in cells distant from the cells where dsRNA is initially introduced^6^,^7^. This phenomenon is referred to as systemic RNAi, and its underlying mechanism may involve intercellular transport of silencing RNA. Although many questions remain to be resolved about the mechanism of systemic RNAi, it is well known that, in *C. elegans*, intercellular RNAi signal, which is assumed to dsRNA^8^, is imported into the cell via a conserved transmembrane protein SID-1, a putative dsRNA transporter^9^,^10^. Meanwhile, endocytosis also plays an important role in cell entry of dsRNA in drosophila S2 cells^11^,^12^,^13^, and the same may be true in *C. elegans*^11^,^14^. In addition, endosome-associated protein, SID-5, is suggested to promote the export of RNAi silencing signals out of the cell^15^. Thus, the importance of membrane traffic in systemic spread of silencing RNA is suggested in *C. elegans*, but little is known about how membrane traffic regulates the phenomenon and which trafficking pathways are involved.

Through a previous genetic screen to search for genes involved in systemic RNAi, namely *rsd* (RNAi Spreading Defective) screen, *rsd-3* alleles have been isolated^16^. *rsd-3* encodes a homolog of epsinR (epsin-related protein), an ENTH domain protein involved in endomembrane trafficking in mammalian cells^17^,^18^. In the previous report, following dsRNA feeding, *rsd-3* mutants showed defects in RNAi in the germline, but were not defective in RNAi in the soma^16^. Thus, RSD-3 is assumed to be a germ cell-specific component of systemic RNAi, and to date, the function of RSD-3 in systemic RNAi is poorly characterized. In the present work, we isolated new alleles of *rsd-3*, and revealed that RSD-3 is involved in systemic RNAi not only in germ cells but also in somatic cells, indicating that RSD-3 is a general component of systemic RNAi. In addition, we found that RSD-3 is required for importing dsRNA into cells rather than exporting from cells. We also found that RSD-3 is associated with endomembranes such as the trans-Golgi network (TGN) and endosomes, and membrane association of the ENTH domain of RSD-3 is required for function. From these results, we suggest that endomembrane trafficking through the TGN and endosomes generally plays an important role in cellular uptake of silencing RNA.

## Results

### RSD-3 is required for systemic RNAi in both germ cells and somatic cells

**Figure 1:**
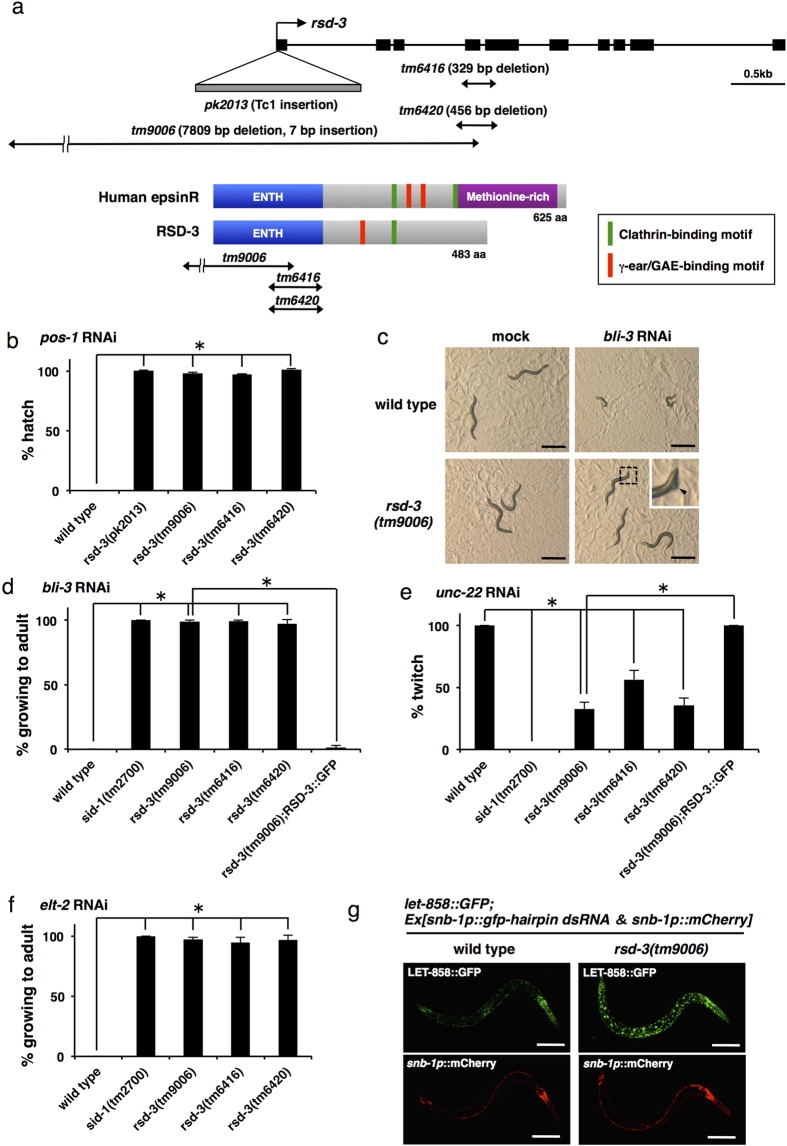
RSD-3 is required for systemic RNAi not only in germ cells but also in somatic cells. (**a**) Upper: genomic structure of *rsd-3*. Black boxes indicate coding exons. Deletion regions of *tm9006*, *tm6416* and *tm6420*, which were isolated in this study, and the Tc1 insertion site in *pk2013* are indicated. Lower: Schematic representation of human epsinR and RSD-3 proteins. Protein domains and motifs are indicated. γ-ear/GAE indicates ear domain of the γ-subunit of AP-1 (adaptor protein-1) and the γ-adaptin ear (GAE) domain of GGA (Golgi-localized, Gamma-ear-containing, Arf-binding protein). AP-1 and GGA are both clathrin adaptors. Corresponding deletion regions of *tm9006*, *tm6416* and *tm6420* are indicated. (**b**) Newly isolated *rsd-3* mutants show resistance to germline RNAi induced by *pos-1* dsRNA feeding as *rsd-3(pk2013)*. Bars represent the percentage of hatched progeny. (**c,d**) *rsd-3* mutants show resistance to *bli-3* feeding RNAi. (**c**) L1 larvae were placed on plates seeded with bacteria without dsRNA (mock) or bacteria expressing *bli-3* dsRNA, and 48 hr later photographed. Wild type animals that were fed on food with *bli-3* dsRNA showed severe molting defects and resulted in larval arrest, whereas almost all *rsd-3(tm9006)* worms reached adulthood. A portion of *rsd-3(tm9006)* worms showed mild cuticle blister formation (inset, arrowhead). Scale bars, 500 μm. (**d**) Bars represent the percentage of animals reached adulthood. Expression of RSD-3::GFP under the control of the *rsd-3* promoter (see Fig. 2a for a schematic representation of the construct) rescues the phenotype of *rsd-3(tm9006)*. (**e**) *rsd-3* mutants show resistance to *unc-22* feeding RNAi. Bars represent the percentage of twitching animals. (**f**) *rsd-3* mutants show resistance to *elt-2* feeding RNAi. Bars represent the percentage of animals reached adulthood. (**g**) *rsd-3(tm9006)* worms show resistance to transgene-induced systemic RNAi. Expression of gfp hairpin RNA and mCherry in the neurons induces silencing of GFP signal (LET-858::GFP) in most cells of wild type animals, but not in *rsd-3(tm9006)* background (upper panels). *snb-1p*::mCherry indicates the neurons that express gfp hairpin RNA (lower panels). Scale bars, 100 μm. Data are shown as mean ± SEM of three separate experiments. *P < 0.001 (Student’s *t*-test, two-tailed).

To understand the role of membrane trafficking in the intercellular transport of silencing RNA in *C. elegans*, we searched for mutants of membrane trafficking-related genes exhibiting defects in systemic RNAi spreading. We assayed various mutants of endocytosis, exocytosis or intracellular membrane trafficking-related genes for their sensitivity to RNAi induced by feeding transformed bacteria expressing dsRNA (feeding RNAi). Among 61 strains examined (Supplementary Table S1), we found that the RB733 strain showed resistance to feeding RNAi. The RB733 strain contains a deletion (*ok498*) in the *ctbp-1* gene, a homolog of C-terminal binding protein 1 (CtBP1), which is involved in macropinocytosis in mammals^19^. We found that the RB733 strain showed resistance to RNAi when fed on dsRNA against both germline expressed gene *pos-1* and soma expressed gene *bli-3*, an epithelial gene (Supplementary Fig. S1). However, other *ctbp-1* mutants (*tm6130* and *tm6188*) did not phenocopy the RB733 strain (Supplementary Fig. S1) and the defects of the RB733 strain were not rescued by expression of *ctbp-1*, suggesting the existence of a mutation other than *ctbp-1(ok498)* in the RB733 strain. We performed whole-genome sequencing of the RB733 strain and identified a large deletion in the *rsd-3* gene. This mutation was confirmed by Sanger sequencing and revealed a 7809 bp deletion and 7 bp insertion located in the genomic region of *rsd-3*. We named this mutation *tm9006* (Fig. 1a). *rsd-3* and *ctbp-1* are both located on chromosome X, 10.9 cM apart from each other. By crossing the RB733 strain with the wild type, we obtained segregants that contained only the *ctbp-1(ok498)* mutation or only the *rsd-3(tm9006)* mutation. Mutants that contained only *rsd-3(tm9006)* were resistant to both *pos-1* and *bli-3* feeding RNAi (Fig. 1b–d), but mutants that contained only *ctbp-1(ok498)* showed a normal response to feeding RNAi (Supplementary Fig. S1b,c), suggesting that the *rsd-3(tm9006)* mutation is essentially responsible for the systemic RNAi defects observed in the RB733 strain.

To examine whether *rsd-3(tm9006)* shows systemic RNAi defects in somatic tissues other than epithelia, where *bli-3* is expressed, we fed *rsd-3(tm9006)* bacteria that express dsRNA targeting the muscle-expressed gene (*unc-22*) and intestine-expressed gene (*elt-2*), and found that *rsd-3(tm9006)* was resistant to *unc-22* and *elt-2* feeding RNAi (Fig. 1e,f). These results suggest that *rsd-3(tm9006)* is resistant to feeding RNAi throughout the organism. However, unlike *sid-1* mutants, that were fully resistant to systemic RNAi, *rsd-3* mutants were partially resistant to silencing soma-expressed genes (Fig. 1c–f).

**Figure 2:**
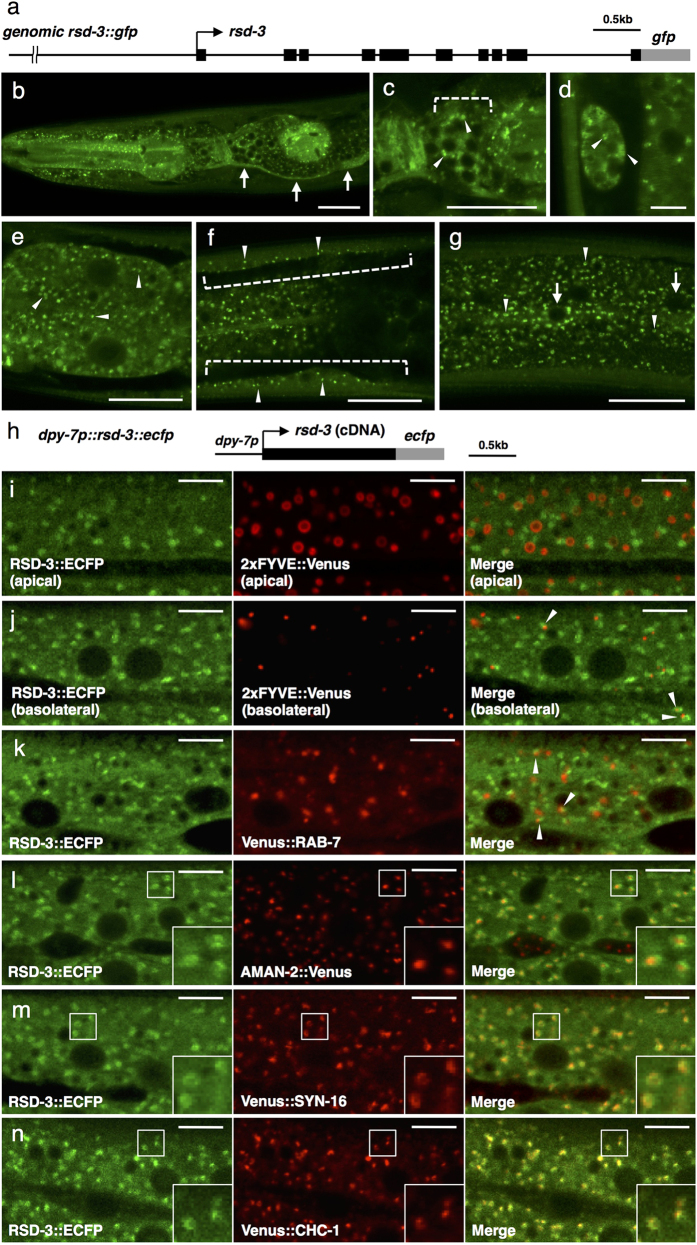
Expression pattern of RSD-3. (**a**) Schematic representation of the *genomic rsd-3::gfp* expression construct. The genomic region of wild type *rsd-3* including 4 kb of upstream promoter sequences and the full-length *rsd-3* was C-terminally fused to *gfp*. Black boxes indicate coding exons of *rsd-3*. Gray box indicates *gfp* sequence. (**b–g**) RSD-3::GFP is ubiquitously expressed. The expression pattern of *genomic rsd-3::gfp* was observed. Arrowheads indicate cytoplasmic puncta. (**b**) Pharynx, head neurons and excretory canal (arrows). (**c**) Head neurons (dotted bracket). (**d**) Coelomocyte. (**e**) Intestine. (**f**) Body wall muscle (dotted brackets). (**g**) Hypodermis and seam cell. Arrows indicate seam cell nuclei. Scale bars in (**b,c,e–g**), 20 μm and scale bar in (**d**), 5 μm. (**h**) Schematic representation of the hypodermis-specific *rsd-3::ecfp* expression construct. *dpy-7* promoter (431 bp) was used as hypodermis-specific promoter. Black box indicates *rsd-3* cDNA. Gray box indicates *ecfp* sequence. (**i–n**) RSD-3::ECFP is associated with the TGN and endosomes in hypodermal cells. ECFP-tagged RSD-3 and Venus-tagged organelle markers were coexpressed under the *dpy-7* promoter and observed in late L4 stage worms. ECFP fluorescence is pseudocolored green, and Venus fluorescence is pseudocolored red. (**i,j**) RSD-3::ECFP does not substantially overlap with the early endosome marker 2xFYVE::Venus both near the apical (**i**) and basolateral (**j**) plasma membranes, but structures labeled by both RSD-3::ECFP and 2xFYVE::Venus are occasionally observed (arrowheads). (**k**) RSD-3::ECFP shows no significant overlap with the late endosome marker Venus::RAB-7, but structures labeled by both RSD-3::ECFP and Venus::RAB-7 are occasionally observed (arrowheads). (**l**) RSD-3::ECFP closely associates with the medial Golgi marker AMAN-2::Venus, but not substantially overlaps each other. Most RSD-3::ECFP formes somewhat ring-like structures, and surrounds the AMAN-2::Venus-positive puncta. (**m,n**) RSD-3::ECFP colocalizes extensively with the TGN marker, Venus::SYN-16 (**m**) and Venus::CHC-1 (**n**). Enlarged images of the boxed areas are shown in the inset (**l–n**). Scale bars, 10 μm.

*rsd-3* encodes a homolog of epsinR, an evolutionarily conserved epsin family protein^20^,^21^. RSD-3/epsinR contains an N-terminal ENTH domain, a well-characterized phosphoinositide binding module^20^,^21^, and motifs for binding clathrin and clathrin adaptor proteins in the C-terminal region (Fig. 1a). In mammalian cells, epsinR has been implicated in clathrin-mediated membrane trafficking between the *trans*-Golgi network (TGN) and endosomes^17^,^18^,^22^,^23^,^24^. Previously, Tijsterman *et al*. performed a forward genetic screen named *rsd* (RNAi Spreading Defective) screen, and *rsd-3* alleles such as *pk2013* were isolated^16^. In their report, following feeding of dsRNA, *rsd-3(pk2013)* showed defects in transmitting the RNAi effect to the germline, but show a normal RNAi response in somatic tissues. We also confirmed the phenotypes of *rsd-3(pk2013)*; i.e., *rsd-3(pk2013)* showed no RNAi phenotype when fed *pos-1* dsRNA, but displayed RNAi phenotype when fed *bli-3* dsRNA (Fig. 1b and Supplementary Fig. S2e). Because *rsd-3(tm9006)* exhibits systemic RNAi defects in both the germline and the soma, we examined whether RSD-3 actually plays a role in systemic RNAi in somatic tissue. We generated a transgene containing *rsd-3* genomic sequences including 4 kb of upstream promoter sequences, the complete ORF and introns, fused in frame to GFP (see Fig. 2a for a schematic representation of the construct), and introduced this transgene into *rsd-3(tm9006)*. We found that expression of RSD-3::GFP completely rescued the silencing defects in the soma of *rsd-3(tm9006)* (Fig. 1d,e). To provide further proof for the gene identity of *rsd-3*, we isolated other *rsd-3* mutants, *tm6416* and *tm6420* (Fig. 1a), and found that these mutants showed systemic RNAi defects not only in the germline (Fig. 1b) but also in the soma (Fig. 1d–f) as was the case with *rsd-3(tm9006)*. From these results, we conclude that RSD-3 is required for systemic RNAi in somatic cells as well as in germ cells.

Because transgene-induced silencing information is also transported between cells^9^, we also examined the response of *rsd-3(tm9006)* to RNAi triggered by a transgene. We generated an RNAi-inducing transgene expressing gfp hairpin RNA and mCherry under the control of the panneuronal *snb-1* promoter and introduced this transgene into *let-858::gfp* transgenic animals, which express nuclear-localized GFP in all somatic cells^25^. In the wild type, neuronally expressed gfp hairpin RNA spread to other tissues and potently silenced the GFP signal in most cells, although it did not affect GFP expression in the neurons as reported previously^26^ (Fig. 1g, upper left panel). In contrast, GFP expression was not prominently silenced in any of the somatic cells with the *rsd-3(tm9006)* background (Fig. 1g, upper right panel). These results indicate that RSD-3 is involved in transport of both environmentally- and transgene-derived silencing signals between cells.

We also investigated why the originally identified *rsd-3(pk2013)* exhibited no defects in systemic RNAi in somatic tissues. In *pk2013*, Tc1 transposon (a 1.6-kb repeated DNA sequence) is inserted within the first exon of *rsd-3*, immediately after the start codon^16^. To determine whether the *pk2013* mutation affects the expression or function of RSD-3 in the soma, we constructed a *pk2013* mutation-containing *rsd-3*, amplified from the *rsd-3(pk2013)* genome, with a C-terminal fusion to GFP under the control of the *rsd-3* promoter (*genomic rsd-3_pk2013::gfp*) (see Supplementary Fig. S2a for a schematic representation of the construct), and generated an extrachromosomal array in *rsd-3(tm9006)*. We detected GFP expression in all somatic tissues, and found that the subcellular localization of the GFP signal was indistinguishable from that of normal RSD-3::GFP (Supplementary Fig. S2b–d and see below). Furthermore, expression of *genomic rsd-3_pk2013::gfp* completely rescued the systemic RNAi defects in the soma of *rsd-3(tm9006)* (Supplementary Fig. S2e). These results indicate that the *pk2013* mutation did not strongly affect RSD-3 expression and function in the soma. As reported for some Tc1 insertions^27^,^28^, in somatic cells of *rsd-3(pk2013)*, Tc1 may be excised from the genome, or may be removed from pre-mRNA by splicing, resulting in the production of an in-frame message to produce RSD-3 protein that is functional *in vivo*.

We tested this hypothesis by analyzing the sequence of *rsd-3* cDNA derived from the Tc1-containing allele of *rsd-3* in somatic cells. We used an extrachromosomal array of *genomic rsd-3_pk2013::gfp* described above, because transgene expression from an extrachromosomal array is typically restricted to somatic cells^25^. mRNA from the wild type animals, *rsd-3(tm9006)* and *rsd-3(tm9006);Ex[genomic rsd-3_pk2013::gfp]* were reverse transcribed, and *rsd-3* cDNAs around the Tc1 insertion site were PCR amplified (see Supplementary Fig. S3a,b). As shown in Supplementary Fig. S3b, no PCR product was observed from *rsd-3(tm9006)* as expected. The size of the PCR product from *rsd-3(tm9006);Ex[genomic rsd-3_pk2013::gfp]* was almost the same as that from wild type, suggesting that most of Tc1 is spliced from mature mRNA of *genomic rsd-3_pk2013::gfp* or imprecisely excised from the extrachromosomal array (Supplementary Fig. S3b). Then, we cloned the PCR product from *rsd-3(tm9006);Ex[genomic rsd-3_pk2013::gfp]* and sequenced five independent cDNA clones (#1~5). As shown in Supplementary Fig. S3c, of the five cDNA clones, three clones (#1~3) have 48 bp insertion sequences at the site corresponding to genomic Tc1 insertion site. These insertions do not lead to premature stop codons, and thus, the three clones maintain the normal *rsd-3* translational reading frame. The predicted amino acid sequences translated from such mRNAs contain extra 16 amino acids at the site 11 amino acids downstream from the N-terminus. This insertion site is located upstream of the functionally important region of the ENTH domain (see below), and therefore, such insertion may not significantly affect the function of RSD-3. Meanwhile, two clones (#4 and #5) have a small deletion and/or insertion, resulting in a frameshift with a premature stop codon (Supplementary Fig. S3c). These results suggest that the Tc1-containing allele of *rsd-3* produces functional RSD-3 in addition to some non-functional RSD-3 in somatic cells.

### RSD-3 is ubiquitously expressed

RSD-3 was reported to be widely expressed in transgenic lines expressing RSD-3::GFP under its own promoter, but the detailed expression pattern was not described^16^. To determine the precise tissue expression, we observed the transgenic animals described above, expressing RSD-3::GFP driven by 4 kb of *rsd-3* upstream sequences (Fig. 2a). Considering the *rsd-3* rescue activity (Fig. 1d,e), expression pattern of the tagged RSD-3 protein very likely reflects those of endogenous protein. RSD-3::GFP fusion protein was expressed in all somatic tissues including the pharynx, neurons, excretory canal, coelomocytes, intestine, body-wall muscles, hypodermis and seam cells (Fig. 2b–g). The RSD-3::GFP fusion protein was diffusely distributed in the cytoplasm and also localized in a punctate pattern in most tissues, indicating that RSD-3 associates with some organelles or vesicles (see the next section). Expression of RSD-3 was previously reported to be high in the coelomocytes^16^, but in our observation, the expression level of RSD-3::GFP in coelomocytes was comparable with those in other tissues. The discrepancy may be due to a difference in the length of *rsd-3* upstream sequences used to generate the transgenes.

### RSD-3 is associated with the TGN and endosomes

To examine the subcellular localization of RSD-3, we performed a series of colocalization studies in the hypodermis of transgenic animals expressing ECFP-tagged RSD-3 and a set of Venus-tagged organelle markers under the control of the *dpy-7* promoter (see Fig. 2h for a schematic representation of *rsd-3::ecfp* expression construct). To identify early endosomes, we used phosphatidylinositol-3-phosphate (PI3P)-binding construct 2xFYVE::Venus. Near the apical membrane of hypodermal cells, most 2xFYVE::Venus formed ring-like structures (Fig. 2i, middle panel), whereas near the basolateral membrane, most 2xFYVE::Venus was mainly found in small punctate structures (Fig. 2j, middle panel), suggesting that apical early endosomes and basolateral early endosomes are morphologically different. RSD-3::ECFP did not substantially overlap with 2xFYVE::Venus near either the apical or basolateral membranes (Fig. 2i,j), although RSD-3::ECFP occasionally overlapped with, or juxtaposed to the 2xFYVE::Venus-positive puncta, especially near the basolateral membrane (Fig. 2j, arrowheads). Likewise, RSD-3::ECFP did not significantly overlap with a late endosome marker, Venus::RAB-7, but occasionally these proteins were partially colocalized, or localized to adjacent domains in close proximity (Fig. 2k, arrowheads). We next examined RSD-3 for Golgi localization. In the hypodermal cells, the medial Golgi marker alpha-mannosidase II::Venus (AMAN-2::Venus) was targeted to puncta scattered throughout the cell, a pattern consistent with localization of Golgi ministacks in most invertebrate cells (Fig. 2l, middle panel)^29^. Most RSD-3::ECFP-positive structures were close to, but did not substantially overlap with, AMAN-2::Venus signals (Fig. 2l). More specifically, most RSD-3::ECFP formed somewhat ring-like structures, and surrounded the AMAN-2::Venus-positive puncta (Fig. 2l, insets). As for the *trans*-Golgi network (TGN), almost all RSD-3::ECFP-positive structures colocalized extensively with the TGN marker, Venus::SYN-16^30^ (Fig. 2m). RSD-3::ECFP also strongly colocalized with the Venus-tagged clathrin heavy chain (Venus::CHC-1) (Fig. 2n). A major population of intracellular clathrin has been reported to localize to the TGN^31^, confirming RSD-3 localization to the TGN. Taken together, these results indicate that RSD-3 is mainly localized to the TGN, with a minor portion localized to endosomes.

The subcellular localization of RSD-3 in the coelomocytes was also checked with double labeling of RSD-3::mCherry (see Supplementary Fig. S4a for a schematic representation of the expression construct) with endocytic compartment GFP-markers. RSD-3::mCherry partially overlapped with, or was juxtaposed to the early endosome marker 2xFYVE::GFP, the late endosome marker GFP::RAB-7 and the recycling endosome marker GFP::RME-1 (Supplementary Fig. S4b–d, arrowheads). RSD-3::mCherry also formed ring-like structures surrounding the medial Golgi marker AMAN-2::GFP-positive puncta (Supplementary Fig. S4e, arrowheads), but RSD-3::mCherry did not overlap with the lysosome marker LMP-1::GFP (Supplementary Fig. S4f). These results indicate that the localization pattern of RSD-3 in the coelomocytes is similar to that in the hypodermis. In cultured mammalian cells, human epsinR also localizes primarily to the TGN with a minor portion to the endosomal compartment^17^,^23^. Thus, in addition to the sequence similarity, the subcellular localization of RSD-3 is very similar to that of human epsinR.

### RSD-3 is not an integral part of the RNAi machinery

Because the observed phenotypes of *rsd-3* mutants might be due to disruption of cellular RNAi machinery itself rather than to a systemic RNAi defect, we examined whether RSD-3 plays a role in cell-autonomous RNAi. Tijsterman *et al*. showed that, by injection of *pos-1* dsRNA into one gonad arm, cell-autonomous RNAi machinery is intact in germ cells of *rsd-3(pk2013)*^16^. We also injected various concentrations of *pos-1* dsRNA into both gonad arms of *rsd-3(tm9006)* and found that RNAi efficiency in germ cells of *rsd-3(tm9006)* is comparable to that in the wild type (Supplementary Fig. S5). This result confirmed that RSD-3 is not involved in cell-autonomous RNAi machinery in germ cells, in contrast to RSD-2 and RSD-6, which were also identified in the *rsd* screen^16^ but turned out to be involved in cell-autonomous RNAi machinery, in particular, secondary siRNA amplification^26^.

To examine the involvement of RSD-3 in the RNAi mechanism in somatic cells, we generated an extrachromosomal array expressing gfp hairpin RNA and mCherry under the control of the muscle-specific *myo-3* promoter. This transgene was introduced into *myo-3p::gfp* transgenic animals (*ccIs4251*), which express GFP in the muscle nuclei and mitochondria. In the wild type background, GFP was largely silenced in red muscle cells expressing the GFP-RNAi construct (Supplementary Fig. S6). GFP expression was reduced equally in red muscle cells with an *rsd-3(tm9006)* background (Supplementary Fig. S6), suggesting that the RNAi pathway is not compromised in *rsd-3(tm9006)* muscle cells. These results indicate that RSD-3 is not involved in the RNAi machinery itself, but rather required for the systemic spread of silencing RNA in both somatic and germ cells.

### RSD-3 is involved in the import but not the export of silencing RNA

**Figure 3:**
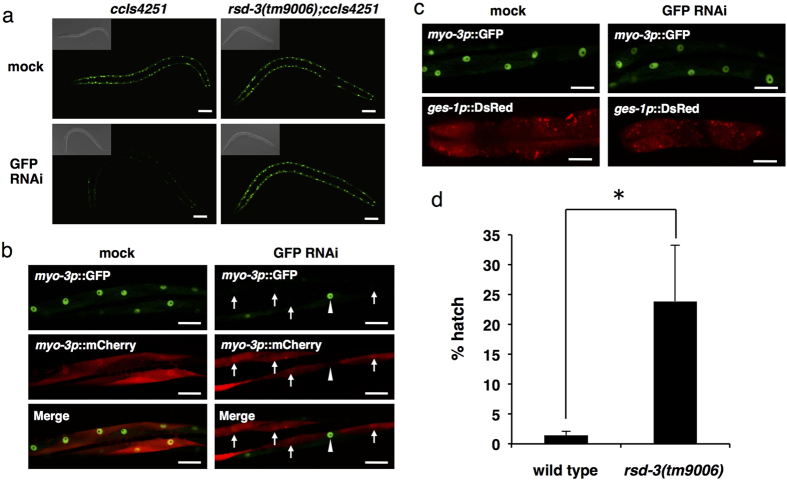
RSD-3 is required for importing silencing RNA into cells. (**a**) *rsd-3(tm9006);ccIs4251* (*myo-3p::gfp*) animals show resistance to GFP feeding RNAi. Body wall muscle GFP fluorescence in *ccIs4251* animals is efficiently reduced by GFP RNAi food (lower left panel), but not in *rsd-3(tm9006);ccIs4251* (lower right panel). Scale bars, 50 μm. (**b**) Body wall muscle-specific expression of RSD-3 restores GFP feeding RNAi sensitivity in body wall muscles of *rsd-3(tm9006);ccIs4251*. *rsd-3(tm9006);ccIs4251;Ex[myo-3p::rsd-3 & myo-3p::mCherry]* worms were fed with mock or GFP RNAi food. *myo-3p*::mCherry indicates the cells expressing RSD-3. Arrows indicate the cells in which RSD-3 is expressed and GFP RNAi is restored. Arrowhead indicates the cell in which RSD-3 is not expressed and GFP RNAi defect is not rescued. Scale bars, 20 μm. (**c**) Intestine-specific expression of RSD-3 does not restore GFP feeding RNAi sensitivity in body wall muscles of *rsd-3(tm9006);ccIs4251*. *rsd-3(tm9006);ccIs4251;Ex[ges-1p::rsd-3 & ges-1p::DsRed]* worms were fed with mock or GFP RNAi food. DsRed fluorescence confirms the expression of *rsd-3* in the intestine (lower panels). Scale bars, 20 μm. (**d**) *rsd-3(tm9006)* animals show partial resistance to pseudocoelomic injection of *pos-1* dsRNA. *pos-1* dsRNA (10 ng/μl) were injected into the pseudocoelom of more than twenty wild type and *rsd-3(tm9006)* animals, and percentage of hatched progeny was scored for each injected animal. Data is shown as mean ± SEM. *P < 0.05 (Student’s *t*-test, two-tailed).

When *C. elegans* is fed with dsRNA, the dsRNA is first taken up by intestinal cells from the intestinal lumen. The silencing RNA is then exported from intestinal cells to the pseudocoelom and subsequently imported into the germ and somatic cells^32^. To determine which step of feeding RNAi is impaired in *rsd-3(tm9006)*, we performed tissue-specific rescue experiments. We used *myo-3p::gfp* transgenic animals (*ccIs4251*) and fed them with bacteria expressing GFP dsRNA. In the wild type background, GFP expression in muscle cells was significantly decreased, but in muscle cells with an *rsd-3(tm9006)* background, GFP was not silenced prominently after exposure to GFP dsRNA (Fig. 3a). We examined whether expression of *rsd-3* cDNA in the body wall muscle or in the intestine rescues the feeding RNAi defect caused by *rsd-3(tm9006)*. We found that expression of RSD-3 and mCherry under the control of the muscle-specific *myo-3* promoter efficiently restored GFP feeding RNAi sensitivity in muscle cells of *rsd-3(tm9006);ccIs4251* animals (Fig. 3b). Especially, GFP was silenced in RSD-3(+) red muscle cells (Fig. 3b, arrows) but not in RSD-3(-) non-red muscle cells (Fig. 3b, arrowhead). On the other hand, feeding RNAi defect was not rescued in muscle cells of *rsd-3(tm9006);ccIs4251* expressing RSD-3 and DsRed under the control of the intestine-specific *ges-1* promoter (Fig. 3c). These results indicate that RSD-3 is not required for the first uptake of silencing RNA into intestinal cells from the intestinal lumen or export from intestinal cells to the pseudocoelom, but rather is required for subsequent import into each cell from the pseudocoelom. To confirm this conclusion, we injected *pos-1* dsRNA (10 ng/μl) into the pseudocoelom. In wild type animals, the injection induced efficient RNAi in the germline, and resulted in almost complete embryonic lethality in the F1 embryos (Fig. 3d). In contrast, *rsd-3(tm9006)* animals were partially resistant to the injection (Fig. 3d). This result supports the view that RSD-3 is required for the import of silencing RNA into cells from the pseudocoelom.

### The ENTH domain is necessary and sufficient to rescue the systemic RNAi defects of *rsd-3* mutants

**Figure 4:**
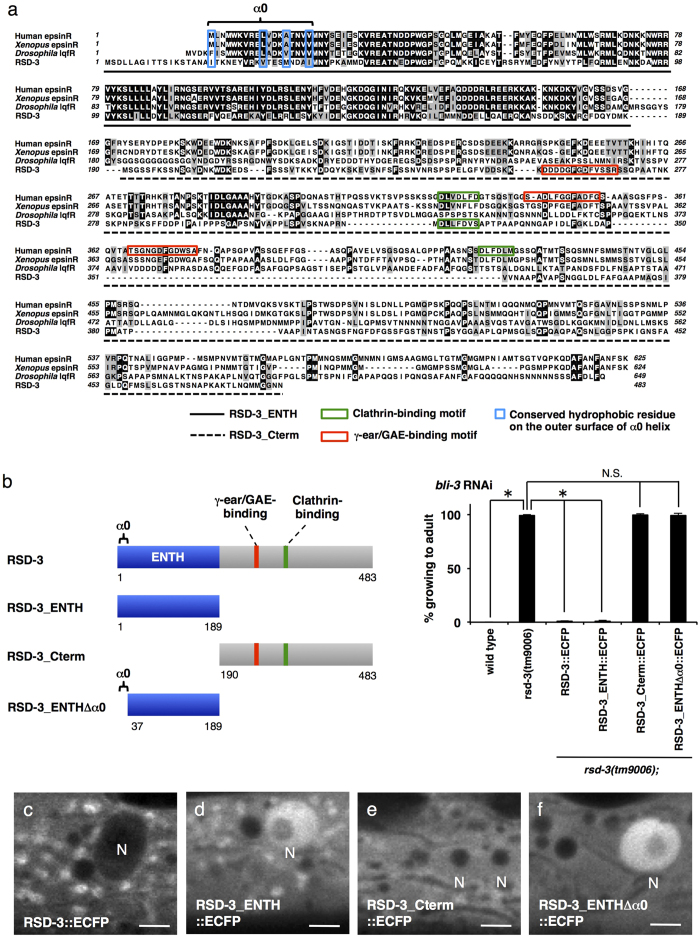
ENTH domain is necessary and sufficient for the function of RSD-3. (**a**) Alignment of the amino acid sequences of RSD-3 and their homologs. The sequences of RSD-3, human epsinR, *Xenopus* epsinR and *Drosophila* lqfR (liquid facets-Related) were aligned using the program ClustalW. Identical amino acids are shown on a black background, and similar amino acids are on a gray background. The solid underline indicates the region corresponding to the ENTH domain of RSD-3 (RSD-3_ENTH), and the dotted underline indicates the region corresponding to the C-terminal region of RSD-3 (RSD-3_Cterm). The region corresponding to putative helix 0 (α0) of human epsinR is indicated with a bracket. Blue boxes indicate conserved hydrophobic residues on the outer surface of α0 helix. Green boxes indicate clathrin-binding motifs of human epsinR and RSD-3. Red boxes indicate γ-ear/GAE-binding motifs of human epsinR and RSD-3. Accession numbers for the sequences used were as follows: human epsinR: NP_055481; *Xenopus* epsinR: NP_001088040; *Drosophila* lqfR: NP_732734; RSD-3: NP_509973. (**b**) Left: schematic representations of RSD-3 truncation constructs. The region containing putative α0 is indicated with a bracket. Amino acid numbers are indicated. Right: ECFP-tagged each construct was expressed in the hypodermis of *rsd-3(tm9006)* and examined the ability to restore sensitivity to *bli-3* feeding RNAi. Bars represent the percentage of animals reached adulthood. Expression of full-length RSD-3 or RSD-3_ENTH fully restores the phenotype in *rsd-3(tm9006)* but RSD-3_Cterm or RSD-3_ENTHΔα0 doesn’t have rescue activity. Data is shown as mean ± SEM of three separate experiments. *P < 0.001 (Student’s *t*-test, two-tailed). N.S.: not statistically different. (**c–f**) Intracellular distribution of ECFP-tagged each construct in the hypodermis. N indicates the nucleus. Full-length RSD-3::ECFP and RSD-3_ENTH::ECFP are localized to puncta (**c,d**). Nuclear localization of RSD-3_ENTH::ECFP is also observed (**d**). RSD-3_Cterm::ECFP is very weakly localized to puncta, and nuclear localization is observed (**e**). RSD-3_ENTHΔα0::ECFP rarely associates with puncta, but nuclear localization is observed (**f**). Scale bars, 5 μm.

Human epsinR possesses an N-terminal ENTH domain with affinity for membrane phosphoinositides, especially for phosphatidylinositol 4-phosphate (PI4P) that is mainly generated on the TGN membrane (Fig. 1a)^17^,^24^. The unstructured C-terminal region of human epsinR contains motifs for binding clathrin and clathrin adaptor proteins, such as AP-1 (adaptor protein-1) and GGAs (Golgi-localized, Gamma-ear-containing, Arf-binding proteins), functioning at the TGN and endosomes (Fig. 1a)^33^,^34^,^35^. These domain/motifs have been proposed to target human epsinR to the TGN and endosomes. In addition, the ENTH domain of human or rat epsinR interacts with specific endosomal soluble NSF attachment protein receptors (SNAREs)^36^,^37^,^38^. Thus, mammalian epsinR has been implicated in clathrin-mediated membrane trafficking between endosomes and the TGN, and particularly functions in sorting of cargoes into transport vesicles^36^,^37^,^39^. RSD-3 possesses domain and motif structures similar to those of human epsinR, except for the methionine-rich domain of unknown function at the extreme C-terminus of human epsinR (Fig. 1a). A sequence alignment of RSD-3 and several epsinR-related proteins shows that the ENTH domain of RSD-3 displays high sequence conservation, whereas the overall sequence conservation in the C-terminal region of RSD-3 is relatively low (Fig. 4a).

As a step toward understanding the role of RSD-3 in systemic RNAi, we examined which parts of RSD-3 are required for restoring feeding RNAi sensitivity of *rsd-3(tm9006)*. We generated expression plasmids for ECFP-tagged full-length RSD-3 and RSD-3 truncations, namely the ENTH domain (RSD-3_ENTH; amino acids 1-189) and the C-terminal region (RSD-3_Cterm; amino acids 190-483) (Fig. 4b). We expressed these constructs separately in the hypodermis of *rsd-3(tm9006)* and examined the ability to restore sensitivity to *bli-3* feeding RNAi. We also observed the distribution of these constructs in hypodermal cells. Expression of full-length RSD-3::ECFP in the hypodermis completely rescued the *bli-3* feeding RNAi defects of *rsd-3(tm9006)* (Fig. 4b), and RSD-3::ECFP fluorescence was observed in a punctate pattern (Fig. 4c). Also, expression of RSD-3_ENTH::ECFP alone fully rescued the defects of *bli-3* feeding RNAi in *rsd-3(tm9006)* (Fig. 4b). RSD-3_ENTH::ECFP had a relatively diffuse distribution in hypodermal cells, and also localized in the nucleus, but still localized in puncta just like full-length RSD-3::ECFP (Fig. 4d). In contrast, expression of RSD-3_Cterm::ECFP could not rescue the phenotype of *rsd-3(tm9006)* at all (Fig. 4b), and in hypodermal cells, while a small portion of RSD-3_Cterm::ECFP was localized in the puncta, most of it was distributed diffusely in the cytoplasm and nucleus (Fig. 4e). These results indicate that the ENTH domain is necessary and sufficient for RSD-3 function in systemic RNAi. It should be noted that, although the C-terminal region has some contribution to RSD-3 localization, only the ENTH domain can be sufficient for targeting RSD-3 to the appropriate subcellular compartments.

The region at the N-terminus of mammalian ENTH domain, which is unstructured when not bound to the lipid ligand, folds an α-helix referred to as helix 0 (α0) upon binding to phosphoinositide^17^,^40^,^41^,^42^. This additional helix is inserted between the lipid head groups due to the hydrophobic residues exposed on the outer surface of helix, and therefore, α0 is required for lipid interaction of mammalian ENTH domain^40^,^41^. The N-terminal region of RSD-3 ENTH domain also contains several hydrophobic residues at the positions corresponding to the outer surface of α0 helix (Fig. 4a, blue boxes), suggesting that the role of α0 is conserved in *C. elegans* RSD-3. Meanwhile, mammalian ENTH domain proteins are thought to have other functions not directly related to membrane trafficking, such as transcriptional regulation in the nucleus^43^. Then, to examine whether membrane interaction is required for the function of RSD-3 ENTH domain in systemic RNAi, we performed rescue experiments with ECFP-tagged RSD-3_ENTHΔα0 (amino acids 37-189), in which the putative α0 was deleted (Fig. 4b). As expected, RSD-3_ENTHΔα0::ECFP rarely associated with puncta, but instead uniformly distributed in the cytosol and the nucleus in hypodermal cells (Fig. 4f), confirming that this truncation mutant cannot interact with membrane. We found that expression of RSD-3_ENTHΔα0::ECFP could not restore *bli-3* feeding RNAi sensitivity of *rsd-3(tm9006)* at all (Fig. 4b). This result strongly suggests that RSD-3 functions in systemic RNAi by regulating membrane trafficking.

## Discussion

Our data indicates that endomembrane-associated RSD-3 generally plays an important role in cellular uptake of silencing RNA. It is known that plasma membrane-localized ENTH domain proteins, such as epsin1/EPN-1, regulate clathrin-mediated endocytosis^21^,^44^, whereas the TGN/endosome-localized epsinR regulates clathrin-mediated intracellular membrane trafficking. Because RSD-3 appears to be an ortholog of human epsinR, it is conceivable that RSD-3 is involved in intracellular membrane trafficking rather than endocytosis. Both human epsinR and Ent3p, an epsinR ortholog in *Saccharomyces cerevisiae*, are reported to be involved in retrograde transport from endosomes to the TGN^18^,^45^, thus RSD-3 may also function in this transport pathway. In addition, the systemic RNAi defects in *rsd-3* mutants were partial, especially in the soma, indicating the existence of functionally redundant genes or pathways.

The ENTH domain, which lacks clathrin and clathrin adaptor protein-binding motifs, is necessary and sufficient for RSD-3 function in systemic RNAi, suggesting that RSD-3 function is not mediated by formation of clathrin-coated vesicles. Instead, because the ENTH domain of mammalian epsinR/Ent3p interacts with some SNAREs such as Vti1b/Vti1p and functions as a cargo adaptor^36^,^37^,^38^,^39^,^46^, RSD-3 may also function as a cargo adaptor for transporting some cargoes to the appropriate compartment. We generated mutants of *vti-1*, a homolog of Vti1b/Vti1p, but *vti-1* mutants did not show systemic RNAi defects (our unpublished observation). Other SNAREs may function redundantly with *vti-1*.

The mechanism by which RSD-3 is involved in the uptake of silencing RNA into cells remains unclear. At present, we suggest the following two possibilities. First, RSD-3 may regulate SID-1 localization. Several plasma membrane transporters, such as GLUT4 (glucose transporter) and Fet3p/Ftr1p (iron transporter), are known to cycle between the plasma membrane and the TGN, via endosomes^47^,^48^. SID-1, which is considered as a plasma membrane-localized dsRNA transporter, may also cycle between the plasma membrane and intracellular compartments via retrograde transport from endosomes to the TGN, and RSD-3 may be involved in this transport pathway and required for the proper localization of SID-1. Second, RSD-3 may regulate the intracellular transport of endocytosed silencing RNA. In this case, SID-1 may transport silencing RNA into the cytosol at an intracellular compartment but not at the plasma membrane. In fact, SID-1::GFP was observed at intracellular compartments as well as the cell periphery^9^. In addition, endomembrane localization of SIDT1 and SIDT2, mammalian homologs of SID-1, was reported^49^,^50^. Growing evidence indicates that RNA silencing is connected to the endomembrane system^2^. Particularly, exogenous siRNAs are loaded into RNA-induced silencing complexes (RISCs) at the cytosolic membrane surface of the rough endoplasmic reticulum (ER)^51^. Thus, it is conceivable that endocytosed silencing RNA arrives at the appropriate compartment, such as ER, by RSD-3-mediated retrograde trafficking, then enters the cytosol through SID-1, and is immediately incorporated to the RNAi machinery. Such a mechanism may be important for the efficient transfer of exogenously delivered silencing RNA into the intracellular RNAi machinery.

Oligonucleotides hold outstanding promise as potential therapeutic agents, but a major concern is their poor accessibility to the targets within the cells. Recently, some studies using mammalian cells suggested that the uptake and trafficking pathway of oligonucleotide could affect the ultimate pharmacological effectiveness^52^,^53^,^54^. Thus, future studies to elucidate the role of RSD-3 in the trafficking of silencing RNA may help to develop an efficient drug delivery system in oligonucleotide therapeutics.

## Methods

### General methods and strains

Bristol N2 was used as wild type strain. Worm cultures, genetic crosses, and other *C. elegans* methods were performed according to standard protocols^55^ except where otherwise indicated. All experiments were performed at 20 °C. The following deletion mutant strains were obtained by TMP (trimethylpsoralen)/UV method^56^: *abt-6(tm5404), aex-3(tm5659), arf-6(tm1447), arl-3(tm1703), arl-6(tm2622), ctbp-1(tm6130)*, *ctbp-1(tm6188)*, *mtcu-1(tm5041), rab-6.1(tm2124), rab-8(tm2526), rab-10(tm2992), rab-11.1(tm2287), rab-11.2(tm2081), rab-14(tm2095), rab-18(tm2121), rab-19(tm2629), rab-21(tm2999), rab-27(tm2270), rab-28(tm2636), rab-30(tm2653), rab-33(tm2641), rab-35(tm2058), rab-37(tm2089), rab-39(tm2466), rsd-3(tm6416)*, *rsd-3(tm6420)*, *scrm-2(tm650), sec-22(tm4552), sid-1(tm2700)*, *snx-1(tm847), snx-3(tm1595), snx-6(tm3790), syx-17(tm3181), tat-1(tm3117), tat-2(tm2332), C52B11.5(tm3007), C56E6.2(tm3008), F11A5.3(tm2585), F11A5.4(tm2567), K02E10.1(tm2564), T04C9.1(tm5548), T28D6.6(tm5550), 4R79.2(tm2640)*. The following mutants and transgenic animals were obtained from the *Caenorhabditis* Genetics Center (CGC, Minneapolis, MN): *arf-1.2(ok796), cav-1(ok2089), cav-2(hc191), ctbp-1(ok498)* (RB733), *efsc-1(ok2572), evl-20(ok1819), exoc-7(ok2006), exoc-8(ok2523), max-2(ok1904), pak-1(ok448), pak-2(ok332), pkc-2(ok328), rab-7(ok511), rap-2(gk11), rsd-3(pk2013)*, *sdpn-1(ok1667), sec-8(ok2187), src-2(ok819), ssr-2(ok1375), tufm-2(ok2850), vps-35(hu68), ccIs4251[myo-3p::gfp::LacZ::NLS & myo-3p::mitochondrial gfp]*, *cdIs39[pcc1::gfp::rme-1(271alpha1)]*, *cdIs54[pcc1::mans::gfp]*, *cdIs66[pcc1::gfp::rab-7]*, *cdIs85[pcc1::2xFYVE::gfp]*, *pkIs1582[let-858::gfp]*, *pwIs50[lmp-1::gfp]*. *rsd-3(tm9006)* was identified as described in “*Identification of rsd-3(tm9006)”* section and isolated by crossing RB733 strain with wild type. Mutations were outcrossed at least five times before further analysis except where otherwise indicated.

### Identification of *rsd-3(tm9006)*

RB733 genomic DNA was purified by phenol and chloroform extraction including RNase A treatment. 1 μg DNA was subjected to fragmentation and adaptor ligation using an Ion Xpress Plus Fragment Library Kit (Life Technologies), according to the manufacturer’s instructions. The library was subjected to emulsion PCR using the Ion OneTouch 200 Template Kit v2 DL (Life Technologies), followed by bead enrichment. Then, whole-genome sequencing was performed with an Ion Torrent PGM system using the Ion PGM 200 Sequencing Kit and Ion 318 chip (Life Technologies). Using IGV (Integrative Genomics Viewer), we inspected the genome region where no read was aligned as the potential deletion candidates. Except for *ok498*, we found two potential deletions larger than 1 kb on chromosome X. One of the two genomic regions contains no protein-coding gene, thus we focused on another region, around chromosome X: +6.73 cM. This mutation candidate was confirmed by Sanger sequencing and revealed a 7809 bp deletion and 7 bp insertion located in the genomic region of *rsd-3*.

### Constructs and transgenic worms

For each construct, at least three independent transgenic lines were analyzed. To generate *genomic rsd-3::gfp*, the genomic region of *rsd-3* (3969 bp upstream the ATG initiation codon and the full-length *rsd-3*) was amplified by PCR from N2 genomic DNA and inserted into pPD95.75 (a gift from Dr. A. Fire) at the SalI and SmaI sites. *snb-1p::gfp-hairpin* was generated as described previously^26^. In brief, GST-loop was amplified by PCR from pGEX-6P-1 (GE Healthcare) and inserted into pPD49.26 (a gift from Dr. A. Fire) at the SmaI and NheI sites, yielding *pPD49.26_GST-loop*. GFP sense sequence was amplified by PCR from pPD95.67 (a gift from Dr. A. Fire) and inserted into *pPD49.26_GST-loop* at the XbaI and SmaI sites, yielding *pPD49.26_GFPsense_GST-loop*. GFP antisense sequence was amplified by PCR from pPD95.67 and inserted into *pPD49.26_GFPsense_GST-loop* at the NheI and SacI sites, yielding *pPD49.26_GFPsense_GST-loop_GFPantisense*. The promoter region of *snb-1* (3015 bp) was amplified by PCR from N2 genomic DNA and inserted into *pPD49.26_GFPsense_GST-loop_GFPantisense* at the HindIII and PstI sites. To generate *genomic rsd-3_pk2013::GFP*, the genomic region of *rsd-3* (3969 bp upstream the ATG initiation codon and the full-length *rsd-3* containing Tc1 insertion) was amplified by PCR from *rsd-3(pk2013)* genomic DNA and inserted into pPD95.75 at the SalI and SmaI sites. To generate *dpy-7p::rsd-3::ECFP*, *rsd-3* cDNA was amplified by PCR from N2 cDNA and inserted into pPD95.75 at the SalI and SmaI sites, yielding *pPD95.75_rsd-3cDNA*. The promoter region of *dpy-7* (431 bp) was amplified by PCR from N2 genomic DNA and inserted into *pPD95.75_rsd-3cDNA* at the HindIII and SalI sites, yielding *pPD95.75_dpy-7p_rsd-3cDNA*. The ECFP sequence was amplified by PCR from pFX_ECFPT^57^ and substituted GFP with ECFP using the SmaI and EcoRI sites. To generate *dpy-7p::aman-2::Venus*, the Venus sequence was amplified by PCR from pFX_VenusT^57^ and substituted GFP with Venus using the SmaI and EcoRI sites, yielding *pPD95.75_Venus*. The *aman-2* cDNA fragment (1-84aa) was amplified by PCR from N2 cDNA and inserted into pPD95.75 at the SalI and SmaI sites, yielding *pPD95.75_aman-2_Venus*. *dpy-7* promoter was subcloned into *pPD95.75_aman-2_Venus* at the HindIII and SalI sites. To generate *dpy-7p::2xFYVE::Venus*, a dimeric FYVE domain from Hrs (2xFYVE) sequence was amplified by PCR from *cdIs85* genomic DNA and inserted into *pPD95.75_Venus* at the SalI and SmaI sites. Then, *dpy-7* promoter was subcloned at the HindIII and SalI sites. To generate *dpy-7p::Venus::rab-7*, *Venus::rab-7cDNA* sequence was subcloned from *pFX_vha-8p_VenusT/rab-7* (E. Kage-Nakadai *et al*. unpublished) into pPD95.75 at the SalI/SmaI sites. Then, *dpy-7* promoter was subcloned at the HindIII and SalI sites. To generate *dpy-7p::Venus::syn-16*, genomic *syn-16* (1261 bp) was amplified by PCR from N2 genomic DNA and using *dpy-7p::Venus::rab-7*, substituted *rab-7* cDNA with genomic *syn-16* at the BglII and SmaI sites. To generate *dpy-7p::Venus::chc-1*, genomic *chc-1* (5535 bp) was amplified by PCR from N2 genomic DNA and using *dpy-7p::Venus::rab-7*, substituted *rab-7* cDNA with genomic *chc-1* at the BglII and SmaI sites. To generate *unc-122p::rsd-3::mCherry*, the mCherry sequence was amplified by PCR from pFX_mCherry^58^ and substituted GFP with mCherry using the SmaI and EcoRI sites, yielding *pPD95.75_mCherry*. The promoter region of *unc-122* (685 bp) was amplified by PCR from N2 genomic DNA and inserted into *pPD95.75_mCherry* at the HindIII and XbaI sites, yielding *pPD95.75_unc-122p::mCherry*. Genomic *rsd-3* (4743 bp) was amplified by PCR from N2 genomic DNA and inserted into *pPD95.75_unc-122p::mCherry* at the XbaI and SmaI sites. To generate *myo-3p::gfp-hairpin*, the promoter region of *myo-3* (2599 bp) was amplified by PCR from N2 genomic DNA and using *snb-1p::gfp-hairpin*, substituted *snb-1* promoter with *myo-3* promoter at the HindIII and PstI sites. To generate *myo-3p::rsd-3*, *rsd-3* cDNA was amplified by PCR from N2 cDNA and inserted into pPD95.75 at the SalI and SmaI sites, yielding *pPD95.75_rsd-3 cDNA*. The *myo-3* promoter was subcloned at the HindIII and SalI sites. To generate *ges-1p::rsd-3*, the promoter region of *ges-1* (3275 bp) was amplified by PCR from N2 genomic DNA and inserted into *pPD95.75_rsd-3cDNA* at the HindIII and SalI sites. To generate *dpy-7p::rsd-3_ENTH::ECFP*, *rsd-3_ENTH* sequence (1-189 aa) was amplified by PCR and using *dpy-7p::rsd-3::ECFP*, substituted *rsd-3* with *rsd-3_ENTH* at the SalI and SmaI sites. To generate *dpy-7p::rsd-3_Cterm::ECFP*, *rsd-3_Cterm* sequence (190-483 aa) was amplified by PCR and using *dpy-7p::rsd-3::ECFP*, substituted *rsd-3* with *rsd-3_ENTH* at the SalI and SmaI sites. To generate *dpy-7p::rsd-3_ ENTH*Δ*α0::ECFP*, *rsd-3_ ENTH*Δ*α0* sequence (37-189 aa) was amplified by PCR and using *dpy-7p::rsd-3::ECFP*, substituted *rsd-3* with *rsd-3_ ENTH*Δ*α0* at the SalI and SmaI sites. The micro-injection of the DNA constructs described above was performed as previously reported^59^ with appropriate selection markers. The transgenic strains constructed for this study: *tmEx3441[snb-1p::gfp-hairpin & snb-1p::mCherry], tmEx3578[genomic rsd-3::GFP], tmEx3681[genomic rsd-3_pk2013::GFP], tmEx3713[dpy-7p::rsd-3cDNA::ECFP & dpy-7p::aman-2(1*-*84aa)::Venus], tmEx3717[dpy-7p::rsd-3cDNA::ECFP & dpy-7p::Venus::rab-7], tmEx3936[dpy-7p::rsd-3cDNA::ECFP & dpy-7p::Venus::syn-16], tmEx3938[dpy-7p::rsd-3cDNA::ECFP & dpy-7p::2xFYVE::Venus], xhEx3601[dpy-7p::rsd-3cDNA::ECFP & dpy-7p::Venus::chc-1], tmEx3845[unc-122p::rsd-3::mCherry]* (recipient: *cdIs66*)*, tmEx3851[unc-122p::rsd-3::mCherry]* (recipient: *cdIs39*), *tmEx3853[unc-122p::rsd-3::mCherry]* (recipient: *cdIs54*), *tmEx3856[unc-122p::rsd-3::mCherry]* (recipient: *pwIs50*), *tmEx3859[unc-122p::rsd-3::mCherry]* (recipient: *cdIs85*), *tmEx3612[myo-3p::gfp-hairpin & myo-3p::mCherry]*, *tmEx3933[myo-3p::rsd-3 & myo-3p::mCherry]*, *tmEx3750[ges-1p::rsd-3 & ges-1p::DsRed]*, *tmEx3723[dpy-7p::rsd-3_ENTH::ECFP]*, *tmEx3725[dpy-7p::rsd-3_*Cterm*::ECFP], tmEx3615[dpy-7p::rsd-3_ENTH*Δα0*::ECFP]*.

### RNAi experiments

Feeding RNAi was carried out as described^60^. RNAi clones were transformed into *E. coli* HT115(DE3), and the bacteria was grown on 100 μg/mL ampicillin and 1 mM isopropyl-beta-D-thiogalactopyranoside (IPTG). RNAi clones for *pos-1*, *mex-3*, *lin-31*, *bli-3* and *unc-22* were taken from the Ahringer RNAi library (GeneService). RNAi clones for *elt-2* and GFP were generated as following: cDNA fragment of *elt-2* was amplified from N2 cDNA. cDNA fragment of GFP was cut from pPD79.44 using NotI/BglII sites. Then, cDNA fragments of *elt-2* or GFP were cloned into the L4440 (pPD129.36) vector, and transformed *E. coli* HT115 (DE3) with each construct. For *pos-1* and *mex-3* RNAi assay, more than twenty L1 larvae were fed with bacteria expressing *pos-1* or *mex-3* dsRNA, reared to adulthood and allowed to lay eggs for several hours. Then adult animals were removed and 24 hours later, the number of dead eggs and hatched larvae were counted. At least 300 eggs and larva were counted per strain. For *lin-31* RNAi assay, L1 larvae were placed on plates seeded with bacteria expressing *lin-31* dsRNA and 54 hr later, the percentage of animals with Muv (multi-vulva) phenotype was scored. At least 50 animals were counted per strain. For *bli-3* RNAi assay, L1 larvae were placed on plates seeded with bacteria expressing *bli-3* dsRNA and 48 hr later (wild type animals reach adulthood), the percentage of animals that reached adulthood was scored. At least 100 animals were counted per strain. For *unc-22* RNAi assay, L4 larvae were placed on plates seeded with bacteria expressing *unc-22* dsRNA and 48 hr later, the percentage of twitching animals was scored. At least 100 animals were counted per strain. For *elt-2* RNAi assay, L4 larvae were placed on plates seeded with bacteria expressing *elt-2* dsRNA, reared to adulthood and allowed to lay eggs for several hours. Then adult animals were removed and 54 hours later, the percentage of animals that reached adulthood was scored. At least 100 animals were counted per strain. For GFP RNAi, L1 larvae were fed with bacteria expressing GFP dsRNA, reared to L4 larvae and photographed. For injection RNAi experiments, *pos-1* dsRNA was synthesized as following: *pos-1* cDNA fragment attached with T7 promoter at both ends was amplified from N2 cDNA, and *pos-1* dsRNA was transcribed using T7 polymerase (Ambion). Synthesized *pos-1* dsRNA was treated with DNase I, purified by phenol and chloroform extraction, and then injected into both gonad arms or the pseudocoelom of more than twenty day 1 adult hermaphrodites for each strain. The hatching rate of embryos laid between 12 to 24 hr post injection was scored for each injected animal. At least 300 progenies were counted per strain. For transgene-induced RNAi, gfp hairpin RNA was expressed from extrachromosomal arrays and GFP fluorescence was photographed at the L4 stage.

### Sequence analysis of cDNAs derived from *genomic rsd-3_pk2013::gfp transgene*

Total RNA was extracted from mixed-stage *tm9006;tmEx3681[genomic rsd-3_pk2013::gfp]* animals using TRIzol reagent according to the protocol supplied by the manufacturer (Invitrogen). First-strand cDNA was revers transcribed from the total RNA with an oligo-dT primer using SuperScript IV (Invitrogen). *rsd-3* cDNAs in the vicinity of the Tc1 insertion site were PCR amplified using primers shown in Supplementary Fig. S3a. The cDNA amplification products were cloned using TOPO TA Cloning Kit (ThermoFicher Scientific). Five cDNA clones were sequenced in the vicinity of the Tc1 insertion site.

### Microscopy

Worms on NGM (nematode growth medium) plates were imaged with a Leica DFC280 camera using Leica IM500 imaging software. For confocal images, animals in 20 mM azide were mounted on a 5% agar pad on a glass slide. Micrographs were taken on a Zeiss LSM 710 inverted confocal microscope with 488 and 543 lasers and images were processed using ZEN software (Carl Zeiss) except for Supplementary Fig. S6. For Supplementary Fig. S6, micrographs were taken on a Leica TCS SP8 inverted confocal microscope with 488 and 552 lasers and HyD detectors, and images were processed using LAS AF software (Leica).

### Statistical analysis

The standard error of the mean (SEM) was used as the error bar for bar charts plotted from the mean value of the data. All the data were compared using two-tailed Student’s *t*-test. Data were considered significantly different if *P*-values were lower than 0.05.

## Additional Information

**How to cite this article**: Imae, R. *et al*. Endomembrane-associated RSD-3 is important for RNAi induced by extracellular silencing RNA in both somatic and germ cells of *Caenorhabditis elegans*. *Sci. Rep*. **6**, 28198; doi: 10.1038/srep28198 (2016).

## Supplementary Material

Supplementary Information
