# Supplementary material for: Endomembrane-associated RSD-3 is important for RNAi induced by extracellular silencing RNA in both somatic and germ cells of Caenorhabditis elegans
Source: Sci Rep. 2016 Jun 16;6:28198. doi: 10.1038/srep28198 (PMC4910058; doi:10.1038/srep28198)
Supplement: Supplementary Information [file srep28198-s1.pdf]

Supplementary Information

Endomembrane-associated RSD-3 is important for RNAi induced by extracellular silencing RNA in both somatic and germ cells of *Caenorhabditis elegans*

Rieko Imae[1,4], Katsufumi Dejima[1], Eriko Kage-Nakadai[1,5], Hiroyuki Arai[3] and Shohei Mitani[1,2,*]

[1]Department of Physiology, Tokyo Women's Medical University School of Medicine, Tokyo, Japan
[2]Tokyo Women's Medical University Institute for Integrated Medical Sciences, Tokyo, Japan
[3]Graduate School of Pharmaceutical Science, University of Tokyo, Tokyo, Japan
[*]Correspondence: mitani.shohei@twmu.ac.jp
[4]Current address: Graduate School of Pharmaceutical Science, University of Tokyo, Tokyo, Japan
[5]Current address: The OCU Advanced Research Institute for Natural Science and Technology, Osaka City University, Osaka, Japan

Supplementary Table S1. A list of mutants tested for feeding RNAi sensitivity

Strain	Outcrossed	Feeding RNAi defects		Mammalian homolog	Structural and functional description
		Germline[a]	Soma[b]		
cav-1(ok2089)	x5	-	-	Caveolin	Caveolae-dependent endocytosis
cav-2(hc191)	x6	-	+/-	Caveolin	Caveolae-dependent endocytosis
cav-1(ok2089);cav-2(hc191)	x5	+/-	+/-	Caveolin	Caveolae-dependent endocytosis
src-2(ok819)	x5	-	-	Src	Caveolae-dependent endocytosis
pkc-2(ok328)	x5	-	-	PKCα	Caveolae-dependent endocytosis
sdpn-1(ok1667)	x5	-	-	Pacsin2	Caveolae-dependent endocytosis
RB733 *ctbp-1(ok498)*	x5	+	+	CtBP1/BARS	macropinocytosis
pak-1(ok448)	x5	-	-	PAK1	macropinocytosis
pak-2(ok332)	x5	-	-	PAK1	macropinocytosis
max-2(ok1904)	x5	+/-	-	PAK1	macropinocytosis
pak-2(ok332);max-2(ok1904)	x5	+/-	-	PAK1	macropinocytosis
T04C9.1(tm5548)	x5	-	-	GRAF1	CLIC/GEEK endocytic pathway
arf-1.2(ok796)	x5	-	-	ARF1	CLIC/GEEK endocytic pathway
arf-6(tm1447)	x5	-	-	ARF6	Arf6-dependent endocytosis
syx-17(tm3181)	x2	-	-	STX7, STX12	SNARE
sec-22(tm4552)	x5	-	-	SEC22B	SNARE
sec-8(ok2187)[c]	x1	ND	-	EXOC4	exocyst complex
exoc-7(ok2006)	x0	-	-	EXOC7	exocyst complex
exoc-8(ok2523)	x0	-	-	EXOC8	exocyst complex
rab-6.1(tm2124)[c]	x1	ND	-	Rab6	small G
rab-7(ok511)[c]	x1	ND	-	Rab7	small G
rab-8(tm2526)	x2	-	-	Rab8	small G
rab-10(tm2992)	x2	ND	-	Rab10	small G
rab-11.1(tm2287)[c]	x1	ND	-	Rab11	small G
rab-11.2(tm2081)	x2	-	-	Rab11	small G
rab-14(tm2095)	x2	-	-	Rab14	small G
rab-18(tm2121)	x2	-	-	Rab18	small G
rab-19(tm2629)	x2	ND	-	Rab43	small G
rab-21(tm2999)	x2	-	-	Rab21	small G
rab-27(tm2270)	x2	-	-	Rab27	small G
rab-28(tm2636)	x2	-	-	Rab28	small G
rab-30(tm2653)	x0	-	-	Rab30	small G
rab-33(tm2641)	x2	-	-	Rab33	small G
rab-35(tm2058)	x2	-	-	Rab35	small G
rab-37(tm2089)	x2	-	-	Rab37	small G
rab-39(tm2466)	x2	-	-	Rab39	small G
4R79.2(tm2640)	x2	-	-	Rab44	small G
F11A5.3(tm2585)	x0	-	-	Rab2	small G
F11A5.4(tm2567)	x0	-	-	Rab2	small G
K02E10.1(tm2564)	x2	-	-	Rab4, Rab13	small G
C52B11.5(tm3007)	x0	-	-	Rab20	small G
C56E6.2(tm3008)	x0	-	-	Rab5, Rab17	small G
ssr-2(ok1375)	x0	-	-	RASD1, RASD2	small G
rap-2(gk11)	x0	-	-	Rap2	small G
arl-3(tm1703)	x2	-	-	Arl3	small G
arl-6(tm2622)	x0	-	-	Arl6	small G
evl-20(ok1819)[c]	x1	ND	-	Arl2	small G
efsc-1(ok2572)	x0	-	-	SelB	small G
mtcu-1(tm5041)	x0	-	-	GTPBP3	small G
tufm-2(ok2850)[c]	x1	ND	-	TUFM	small G
T28D6.6(tm5550)	x5	-	-	DRG1	small G
aex-3(tm5659)	x0	-	-	MADD	GEF for *rab-3* GTPase
snx-1(tm847)	x5	-	-	SNX1, SNX2	Retromer
snx-3(tm1595)	x4	-	-	SNX3, SNX12	Retromer
snx-6(tm3790)	x4	-	-	SNX6, SNX32	Retromer
vps-35(hu68)	x3	-	-	Vps35	Retromer
tat-1(tm3117)	x5	-	-	ATP8A	phospholipid flippase
tat-2(tm2332)	x5	-	-	ATP8B	phospholipid flippase
tat-1(tm3117);tat-2(tm2332)	x5	-	-	ATP8A, ATP8B	phospholipid flippase
scrm-2(tm650)	x0	-	-	PLSCR1-5	phospholipid scramblase
abt-6(tm5404)	x0	-	-	ABCA subfamily	ABC transporter

-, no apparent defects in feeding RNAi was observed. +, strong defects in feeding RNAi was observed. +/-, mild defects in feeding RNAi was observed. *a*, Assayed by *pos-1* or *mex-3* feeding RNAi. *b*, Assayed by *bli-3* or *lin-31* or *unc-22* feeding RNAi. *c*, Homozygous mutants derived from heterozygous parents were used. ND, not determined.

Supplementary Figure S1

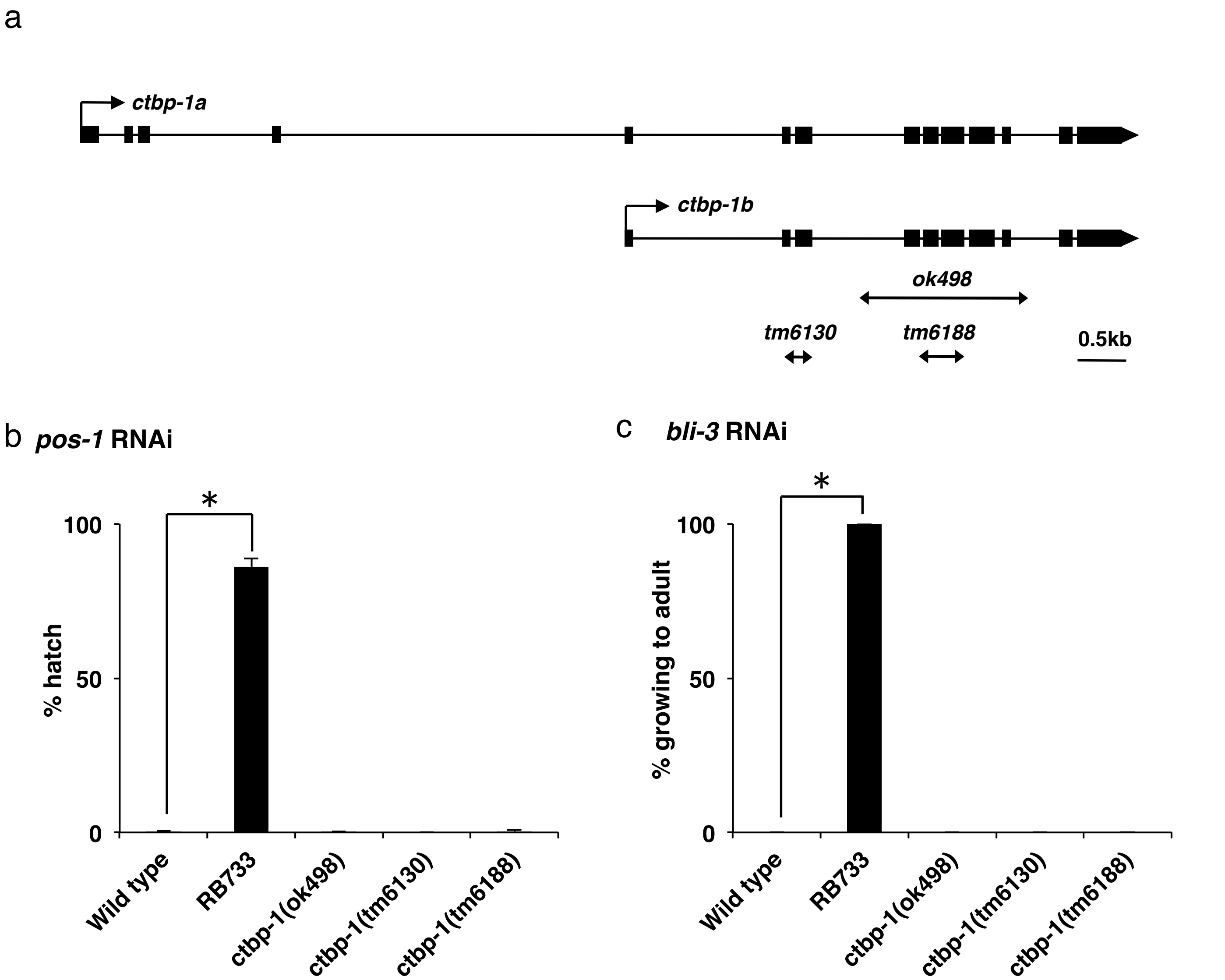

Supplementary Figure S1. Resistance to feeding RNAi in RB733 is not due to the mutation of *ctbp-1*. (a) Genomic structure of *ctbp-1*. Black boxes indicate coding exons. Two isoforms (*ctbp-1a* and *ctbp-1b*) are transcribed from a *ctbp-1* locus. Deletion regions of *ok498*, *tm6130* and *tm6188* are indicated. (b) *pos-1* feeding RNAi. Bars represent the percentage of hatched progeny. (c) *bli-3* feeding RNAi. Bars represent the percentage of animals reached adulthood. RB733 strain containing *ctbp-1(ok498)* shows feeding RNAi defects in both germ and somatic cells, but mutants containing only *ctbp-1* deletion, namely *ok498*, *tm6130* and *tm6188*, respond normally to feeding RNAi in both germ and somatic cells (b, c). Data are shown as mean ± SEM of three separate experiments. $^*P<0.001$ (Student's *t*-test, two-tailed).

Supplementary Figure S2

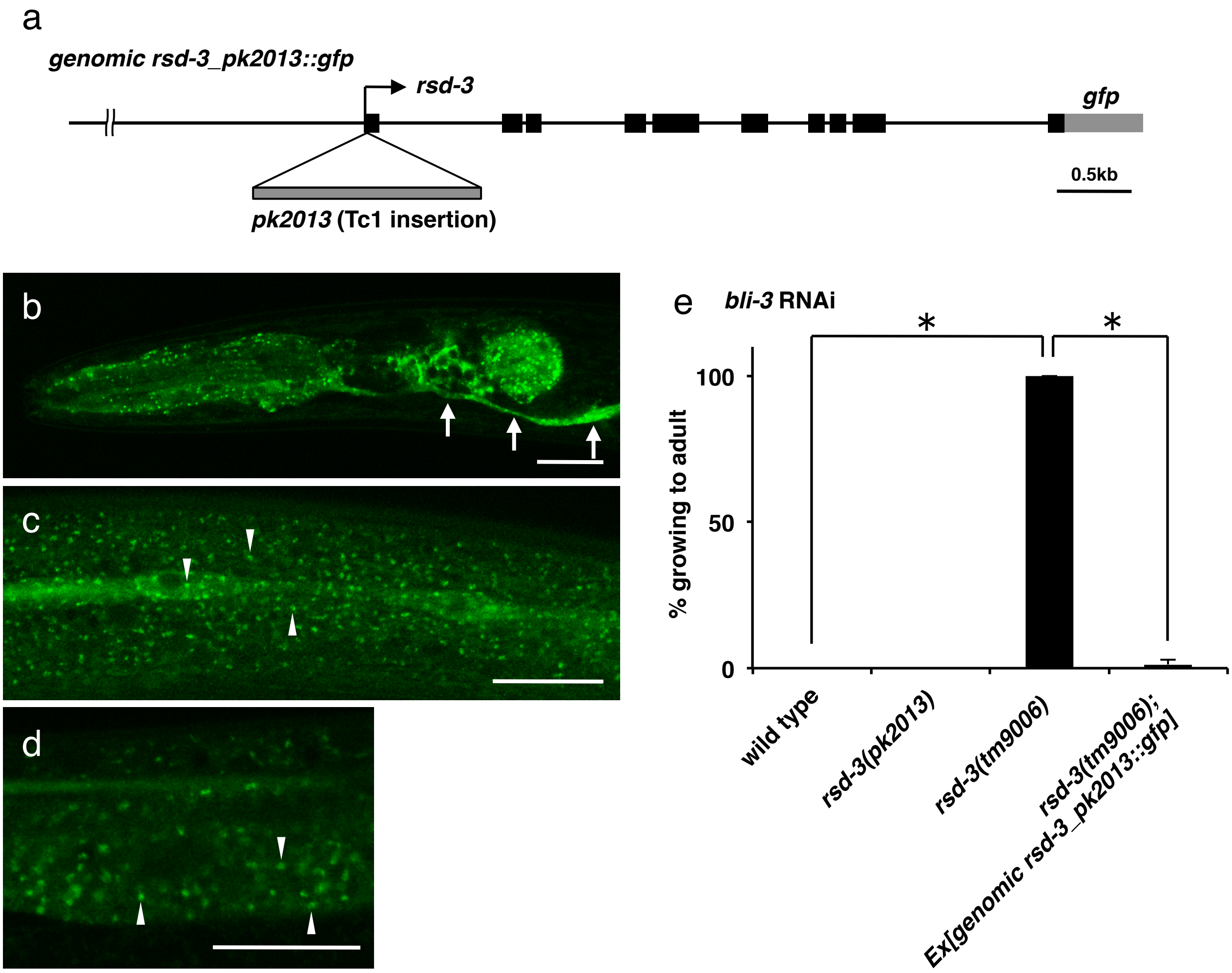

Supplementary Figure S2. Expression and function of RSD-3 are not disrupted by *pk2013* (Tc1 insertion) in somatic cells. (a) Schematic representation of the *genomic rsd-3_pk2013::gfp* expression construct. The genomic region of *rsd-3* including 4 kb of upstream promoter sequences and the full-length *rsd-3* containing Tc1 insertion was amplified from *pk2013* animals and C-terminally fused to *gfp*. Black boxes indicate coding exons of *rsd-3*. (b, c, d) Expression pattern of *genomic rsd-3_pk2013::gfp*. Arrowheads indicate cytoplasmic puncta. (b) Pharynx, head neurons and excretory canal (arrows). (c) Hypodermis and seam cells. (d) Intestine. Scale bars, 20 μm. (e) *bli-3* feeding RNAi. Bars represent the percentage of animals reached adulthood. Expression of *genomic rsd-3_pk2013::gfp* completely restores feeding RNAi sensitivity in the hypodermis of *rsd-3(tm9006)*. Data is shown as mean ± SEM of three separate experiments. *P<0.001 (Student's t-test, two-tailed).

Supplementary Figure S3

a

Tc1 insertion site

```
        10         20         30         40         50         60         70
ATGTCGGACT TGCTAGCCGG AATAACCACA TCTATCAAGA GCACAGCAAA TGCAATCACA AAAAACGAGT

        80         90        100        110        120        130        140
ATGTCCGGAA GGTCACTGAG TCAATGAACG ACGCTATTAT GAATTATCCC AAGGCGATGA TGGACGTTCG

       150        160        170        180        190        200        210
AGAAGCCACC AATGAAGATC CATGGGGACC AACTGGACCA CAAATGAAGA AAATTTGCGA GTATACACGA

       220        230        240        250        260        270        280
TCGAGATATA TGGAGGATTT CTACAATGTC TACACGCCGC TCTTCCAAAG AATGCTGGAA AACAATAAGG

       290        300        310        320        330        340        350
ATGCATGGAG ACGTGTCTAT AAGAGTCTCA TCCTTCTCGA CTACTTGCTA AAAAACGGAA GTGAACGATT

       360        370        380        390        400        410        420
TGTGCAAGAA GCTCGTGAAA AAGCATATGA ACTTCGACGT CTTGAAAGCT ACAAATACAT TGATGAAAAG

       430        440        450        460        470        480        490
GGAAAAGATC AAGGCATTAA CATCCGTCAT CGTGTCAAGC AAATCTTAGA AATGATGAAC GACGACGAGC
```

b

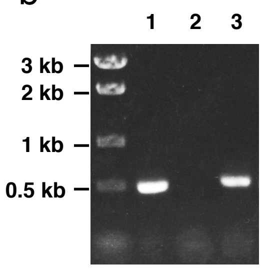

1 wild type
2 *rsd-3(tm9006)*
3 *rsd-3(tm9006); Ex[genomic rsd-3_pk2013::gfp]*

c

Clone	cDNA sequence	Description of change in cDNA sequence	Predicted amino acid sequence
#1, 2	<u>ATG</u>TCGGACTTGCTAGCCGGAATAACCAC<br>ATCTACAGTGCTGGCCAAAAAGATATCCA<br>CTTTTGGTTTTTTCCAGCACTGTATCAAG<br>AGCACAGCAAATGCAATCACAAAAAACGA<br>GTATGTCCGGAAGGTCACTGAGTCAATGA<br>ACGAC…	48 bp insertion which does not lead to premature stop codons.	10 20 30<br><u>M</u>SDLLAGITT STVLAKKIST FGFFQHCIKS<br>40<br>TANAITKNEY VRKVTES...
#3	<u>ATG</u>TCGGACTTGCTAGCCGGAATAACCAC<br>ATCTACAGTGCTGGCCAAAAAGATATCCA<br>CTTTTGGTTTTTTGTGTCACTGTATCAAG<br>AGCACAGCAAATGCAATCACAAAAAACGA<br>GTATGTCCGGAAGGTCACTGAGTCAATGA<br>ACGAC…	48 bp insertion which does not lead to premature stop codons.	10 20 30<br><u>M</u>SDLLAGITT STVLAKKIST FGFLCHCIKS<br>40<br>TANAITKNEY VRKVTES...
#4	<u>ATG</u>TCGGACTTGCTAGCCGGAATAACCAC<br>ATCTACAGTGCTGGCCAAAAAAGATATCC<br>ACTTTTGGTTTTTTCCAGCACTGTATCAA<br>GAGCACAGCAAATGCAATCACAAAAAACG<br>AGTATGTCCGGAAGGTCACTGAGTCAATG<br>AACGAC…	49 bp insertion, resulting in a frameshift with a premature stop codon.	10 20 30<br><u>M</u>SDLLAGITT STVLAKKDIH FWFFPALYQE<br>40<br>HSKCNHKKRV CPEGH* (stop)
#5	<u>ATG</u>TCGGACTTGCTAGCCGGAATAACCAC<br>ATCTACAGTGCTGGCCAAAAAGATATCCA<br>CTTTTGGTTTTTTCAAATGCAATCACAAA<br>AAACGAGTATGTCCGGAAGGTCACTGAGT<br>CAATGAACGAC…	37 bp insertion and 12 bp deletion (35-46 bp), resulting in a frameshift with a premature stop codon.	10 20 30<br><u>M</u>SDLLAGITT STVLAKKIST FGFFKCNHKK<br>RVCPESH* (stop)

Supplementary Figure S3. Sequence analysis of cDNAs derived from *genomic rsd-3_pk2013::gfp* transgene. (a) A portion of *rsd-3* cDNA sequence (1-490 bp). The position corresponding to genomic Tc1 insertion site is indicated. Red arrows indicate the orientation and location of PCR primers for amplifying *rsd-3* cDNAs in the vicinity of the Tc1 insertion site. (b) cDNA PCR products using primers indicated in (a). PCR products of wild type (lane 1), *rsd-3(tm9006)* (lane 2) and *rsd-3(tm9006);Ex[genomic rsd-3_pk2013::gfp]* (lane 3) were separated by electrophoresis. (c) cDNA sequence in the vicinity of the Tc1 insertion site, description of change in cDNA sequence and amino acid sequence predicted from the cDNA sequence of each clone are shown. Inserted cDNA sequences are indicated in red. Predicted extra amino acid sequences are indicated in blue (clone #1~3). Start codon in cDNA sequence and the initiating methionine in predicted amino acid sequence are underlined.

Supplementary Figure S4

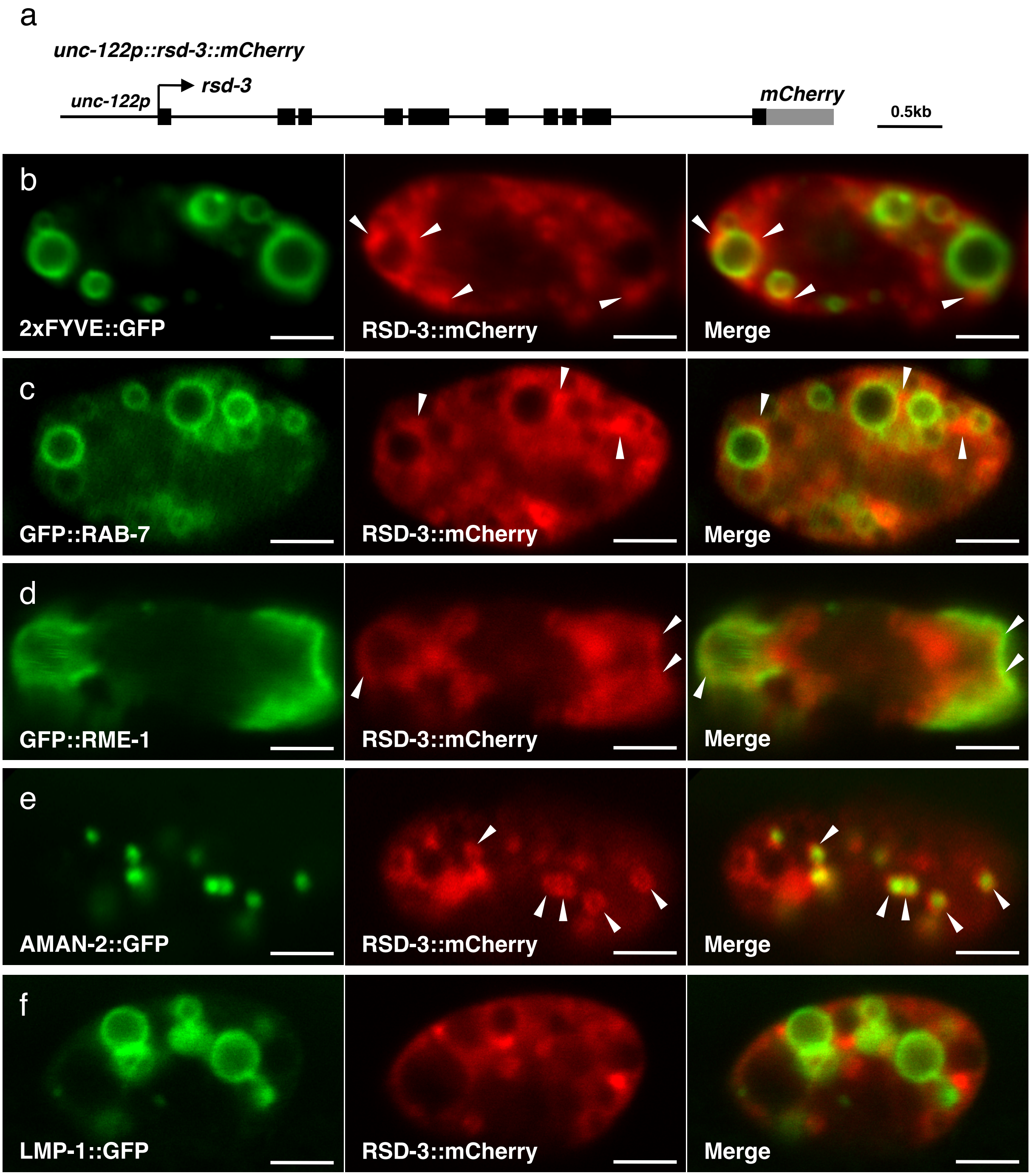

Supplementary Figure S4. Intracellular localozation of RSD-3 in coelomocytes. (a) Schematic representation of the coelomocyte-specific *rsd-3::mCherry* expression construct. *unc-122* promoter (685 bp) was used as coelomocyte-specific promoter. Black boxes indicate coding exons of *rsd-3*. Gray box indicates *mCherry* sequence. (b-f) Confocal images showing RSD-3::mCherry in coelomocytes expressing the indicated GFP fusion markers of early endosomes (2xFYVE::GFP; b), late endosomes (GFP::RAB-7; c), recycling eendosomes (GFP::RME-1; d), medial golgi (AMAN-2::GFP; e) and lysosomes (LMP-1::GFP; f). Arrowheads in (b-d) indicate RSD-3::mCherry closely associated with each organelle marker. Arrowheads in (e) indicate RSD-3::mCherry around medial golgi marker AMAN-2::GFP. Scale bars, 2.5 μm.

Supplementary Figure S5

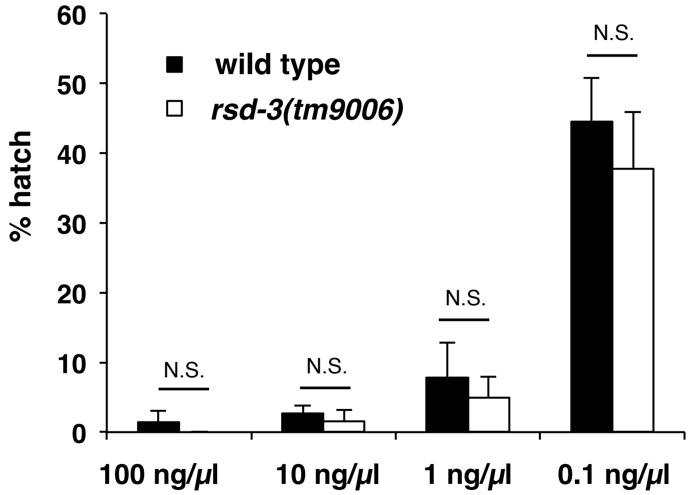

Supplementary Figure S5. RSD-3 is not required for the RNAi machinery itself in germ cells. *pos-1* dsRNA (100, 10, 1, 0.1 ng/μl) were injected into both gonad arms of more than twenty wild type and *rsd-3(tm9006)* animals, and percentage of hatched progeny was scored for each injected animal. Data is shown as mean ± SEM. N.S.: not statistically different.

Supplementary Figure S6

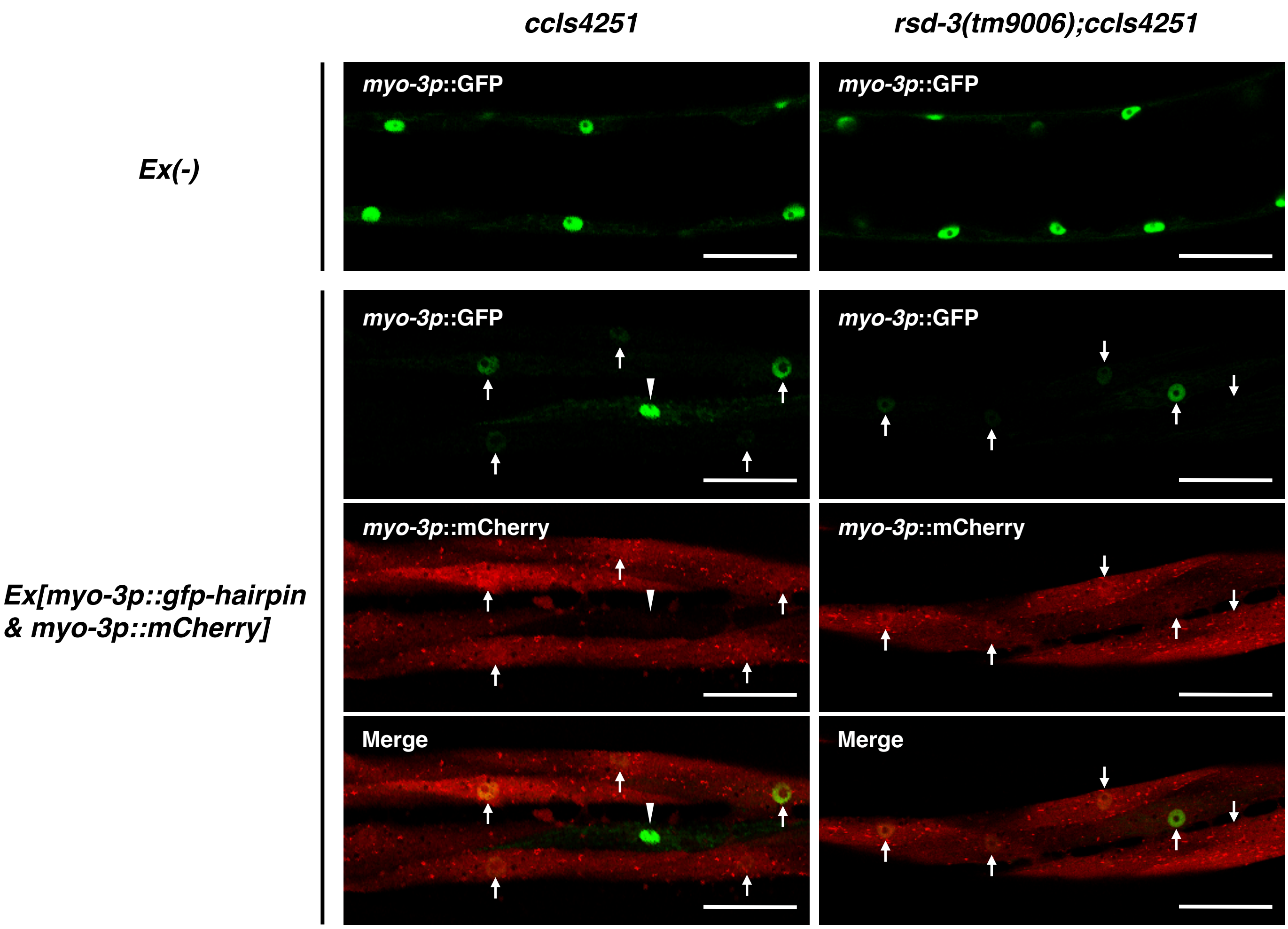

Supplementary Figure S6. RSD-3 is not required for the RNAi machinery itself in somatic cells. Upper: Body wall muscle GFP fluorescence in *ccIs4251* and *rsd-3(tm9006);ccIs4251*. Lower: Expression of gfp hairpin RNA and mCherry in body wall muscles induces silencing of GFP signal (*myo-3p*::GFP) in both *ccIs4251* and *rsd-3(tm9006);ccIs4251*. *myo-3p*::mCherry indicates the cells expressing gfp hairpin RNA. Arrows indicate the cells in which gfp hairpin RNA is expressed. Arrowhead indicates the cell in which gfp hairpin RNA is not expressed. Scale bars, 30 μm.
